# A catalog of metagenomes and metagenome-assembled genomes from culture-enrichment microcosms containing polyethylene as carbon source

**DOI:** 10.1128/mra.00689-24

**Published:** 2024-11-27

**Authors:** Aubrey Dickon Chigwada, Henry Joseph Oduor Ogola, Memory Tekere

**Affiliations:** 1 Department of Environmental Science, College of Agricultural and Environmental Sciences, University of South Africa, Roodepoort, Gauteng, South Africa; Montana State University, Bozeman, Montana, USA

**Keywords:** metagenome-assembled genomes, MAGs, plastic biodegradation, bioremediation, polyethylene, microcosms

## Abstract

We present a data set detailing enrichment microbial consortia degrading polyethylene (PE) plastic. Derived from 180-day microcosm incubations using landfill soil, seawater, and cow dung, the data set includes three metagenomes and 23 metagenome-assembled genomes that capture microbial community interactions, structures, and functions relevant to PE biodegradation.

## ANNOUNCEMENT

The global surge in plastic use has resulted in a significant environmental threat—plastic pollution. Bioremediation has emerged as a promising cost-effective and eco-friendly approach to manage plastic waste (1). While plastic-degrading microbes have been identified, diverse ecosystems likely harbor untapped microbial communities with novel plastic-degrading potential (2). Mixed culture-enriched microcosms from different ecological niches have been shown to outperform single cultures, enhancing plastic degradation through synergistic microbial interactions (3).

To explore this potential, we established 180-day culture-enrichment microcosms using landfill soil (LFS), cow dung (CD), and seawater (SW) in 200 mL minimal salt media (MSM) supplemented with 2 g standard granular PE pellets. Landfill soil was collected from a 14-year-old municipal landfill site in Johannesburg, South Africa (26.1201°S, 27.7870°E), seawater from Durban Harbour (29.8723°S, 31.0249°E), and cow dung from ARC Roodeplaat, Pretoria, South Africa (25.6080°S, 28.3525°E). Triplicate cultures were incubated at 30°C with continuous agitation at 160 rpm, following the method of Roberts et al. (4). The observed degradation rates ranged from 31 ± 0.83 to 34 ± 0.83%. DNA was extracted from 1 mL culture pellet using the Macherey-Nagel NucleoSpin Soil DNA extraction kit (Fisher Scientific UK Ltd., Leicestershire, UK). Replicate DNA samples were pooled, and DNA libraries were prepared using MGIEasy Universal DNA Library Prep Set (MGI Tech Co., Shenzen, China), mixed to create DNA nanoballs, and sequenced on the DNBSEQ-G400 sequencer (MGI Tech Co., Shenzen, China) at Agricultural Research Council, Pretoria, South Africa.

Metagenome sequencing yielded 23,994,198 (LFS), 19,037,958 (CD), and 15,002,358 (SW) paired end reads. Low-quality reads and adapter sequences were removed using Trimmomatic (v0.39) (5), with default settings. The quality reads were then used to perform taxonomic analysis and classification using Kraken2 (v2.1.1) and Bracken (v2.6.2) (6), respectively, with the kraken-filter threshold set at 0.20 using the Standard PlusPF database (https://benlangmead.github.io/aws-indexes/k2). The relative average abundance of identified taxa at kingdom level was 0.65% Archaea, 98.88% Bacteria, 0.66% Eukarya, and 0.004% Viruses.

**Fig 1:**
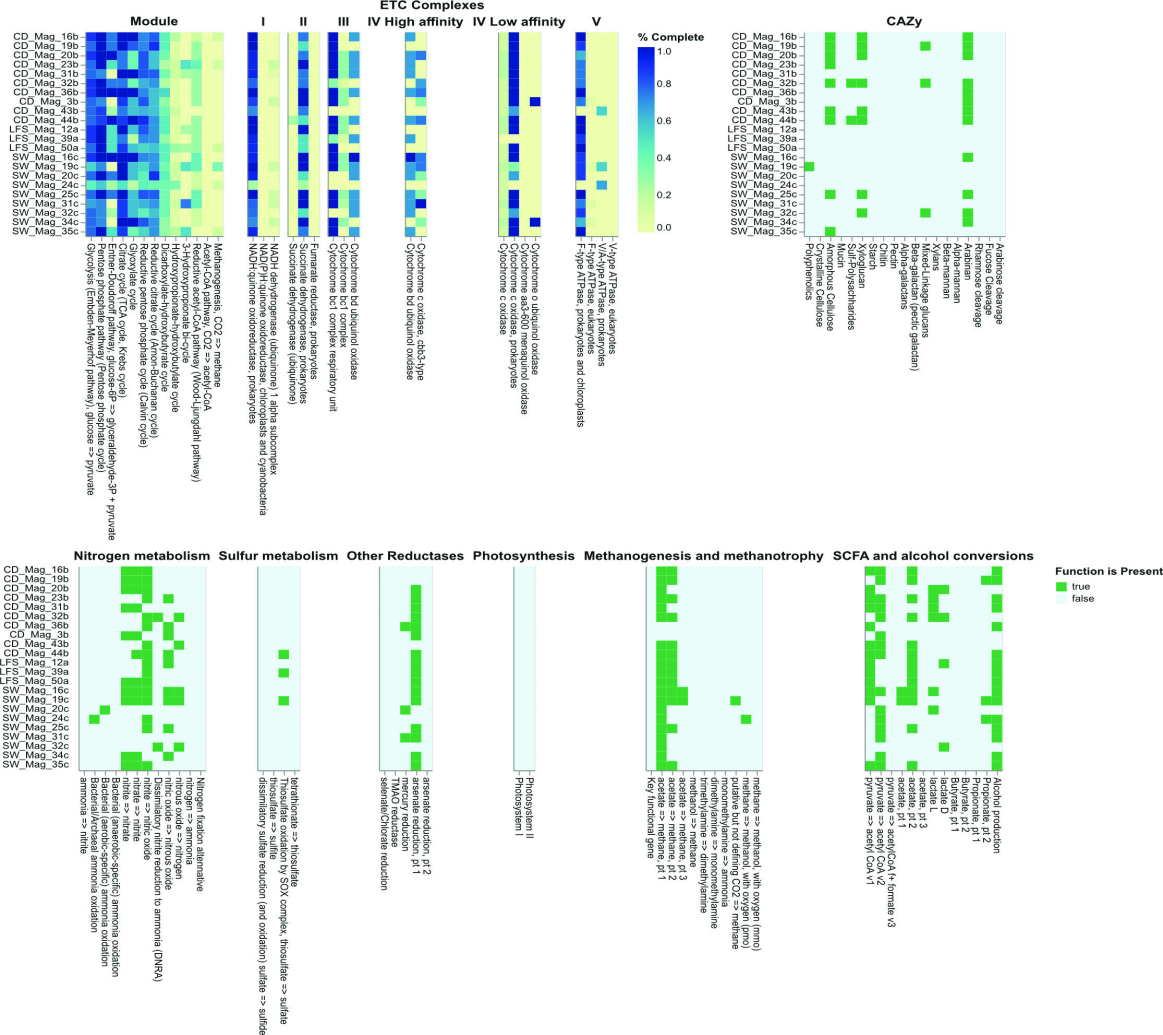
DRAM annotations of the 23 MAGs recovered from the metagenomes of 180-day PE-degrading microcosms.

Quality-filtered reads were assembled using metaSPAdes v3.15.3 (7) with default parameters, producing assemblies of ~384 Mbp in 63,734 contigs. Contig integrity was evaluated with QUAST v5.2.0 (8) and separately binned using MaxBin (v2.0) (9), metaBAT2 (10), and CONCOCT (v1.0.0) (11), using a minimum contig size of 2500 bp. Resultant bins were combined using the DAS tool (v1.1.2) (12) and assessed for quality using CheckM (v1.0.18) (13). Quality (≥50% completeness, ≤10% contamination (14)) and reliable MAGs (<500 contigs and N50 >20,000 bp (15)) were taxonomically annotated with GTDB-Tk (v3.4.2) (16) and Prokka (v1.14.5) (17) and functionally annotated with DRAM v0.1.2) (18), respectively (Table 1; Fig. 1). A total of 23 high-quality MAGs were recovered: 22 Bacteria and 1 Archaea, with 3, 10, and 10 MAGs from LFS, CD, and SW microcosms, respectively (Table 1). Notably, Mag_12 a (*Nitrosomonas*) and Mag_34 c, Mag_31b, and Mag_31 a (Sphingomonadales) are taxa that have been previously implicated in PE biodegradation (19, 20).

**TABLE 1:** Description of 23 MAGs obtained from 180-day enrichment cultures of PE-degrading landfill soil (LFS), cow dung (CD), and seawater (SW) microcosms

Microcosm	MAG	Comp^*a*^ (%)	Cont^*b*^ (%)	Genome size (bp)	Contigs	N50 (bp)	GC (%)	CDS	NCBI Genbank Assembly	GTDB-Tk taxonomy
LFS	Mag_12a	97.63	0	2519992	103	41868	49.7	2167	GCA_040256045.1	d_Bacteria;p_Pseudomonadota;c_Gammaproteobacteria; o_Burkholderiales;f_Nitrosomonadaceae;g_Nitrosomonas;s_
	Mag_39a	96.82	5.76	3566771	196	31103	62.8	3476	GCA_040255845.1	d_Bacteria;p_Nitrospirota;c_Nitrospiria;o_Nitrospirales; f_Nitrospiraceae;g_Nitrospira;s_
	Mag_50a	97.86	1.79	3740453	152	41481	73.4	3632	GCA_040255945.1	d_Bacteria;p_Actinomycetota;c_Thermoleophilia;o_Solirubrobacterales; f_Solirubrobacteraceae;g_Baekduia;s_
CD	Mag_16b	99.74	4.36	7365677	281	45218	71.1	6601	GCA_040255525.1	d_Bacteria;p_Actinomycetota;c_Actinomycetia;o_Mycobacteriales; f_Micromonosporaceae;g_Rugosimonospora;s_
	Mag_19b	99.02	1.34	4127551	76	89253	65.2	3848	GCA_040255675.1	d_Bacteria;p_Actinomycetota;c_Actinomycetia; o_Acidothermales;f_Acidothermaceae;g_;s_
	Mag_20b	89.11	0.61	3526080	192	29573	66.1	3547	GCA_040255805.1	d_Bacteria;p_Pseudomonadota;c_Alphaproteobacteria; o_Rhizobiales;f_Devosiaceae;g_Devosia_A;s_Devosia_A sp023390785
	Mag_23b	98.44	0.43	3109400	15	397146	64.9	3444	GCA_040255145.1	d_Bacteria;p_Pseudomonadota;c_Alphaproteobacteria; o_UBA11222;f_UBA11222;g_;s_
	Mag_31b	89.35	0.88	2008078	156	20276	64.3	2594	GCA_040255785.1	d_Bacteria;p_Pseudomonadota;c_Alphaproteobacteria; o_Sphingomonadales;f_Sphingomonadaceae;g_Allosphingosinicella;s_
	Mag_32b	97.26	2.05	5104168	8	806121	65.1	4049	GCA_040255565.1	d_Bacteria;p_Verrucomicrobiota;c_Verrucomicrobiae; o_Opitutales;f_Opitutaceae;g_Opitutus;s_
	Mag_36b	96.7	0.11	3629533	78	101934	68.8	3183	GCA_040255835.1	d_Bacteria;p_Pseudomonadota;c_Gammaproteobacteria; o_Xanthomonadales;f_Rhodanobacteraceae;g_Rhodanobacter;s_
	Mag_3b	99.13	0.58	2703822	57	76985	69.9	2028	GCA_040255765.1	d_Bacteria;p_Pseudomonadota;c_Alphaproteobacteria; o_Sphingomonadales;f_Sphingomonadaceae;g_Sphingomicrobium;s_
	Mag_43b	89.62	1.86	3216479	163	31456	69.2	3043	GCA_040255665.1	d_Bacteria;p_Deinococcota;c_Deinococci;o_Deinococcales; f_Trueperaceae;g_;s_
	Mag_44b	93.91	3.65	4565572	225	36262	62.4	3897	GCA_040255535.1	d_Bacteria;p_Pseudomonadota;c_Gammaproteobacteria; o_Steroidobacterales;f_Steroidobacteraceae;g_JAAYXG01;s_
SW	Mag_16c	95.34	1.02	3302345	87	62013	66	2910	GCA_040255405.1	d_Bacteria;p_Pseudomonadota;c_Gammaproteobacteria; o_Xanthomonadales;f_Rhodanobacteraceae;g_66–474;s_
	Mag_19c	89.11	1.44	4101367	191	36997	64.1	3751	GCA_040255305.1	d_Bacteria;p_Pseudomonadota;c_Alphaproteobacteria;o_Rhizobiales; f_Hyphomicrobiaceae;g_Hyphomicrobium_C;s_Hyphomicrobium zavarzinii
	Mag_20c	96.59	0	4547147	16	1089700	62.8	3436	GCA_040255465.1	d_Bacteria;p_Planctomycetota;c_Phycisphaerae;o_Phycisphaerales; f_UBA5793;g_UBA5793;s_
	Mag_21c	94.94	7.73	3722808	165	43447	60.4	38	GCA_040255845.1	d_Bacteria;p_Nitrospirota;c_Nitrospiria;o_Nitrospirales;f_Nitrospiraceae;g_;s_
	Mag_24c	96.12	2.91	2550092	182	23823	34.3	2856	GCA_040255245.1	d_Archaea;p_Thermoproteota;c_Nitrososphaeria;o_Nitrososphaerales; f_Nitrososphaeraceae;g_Nitrosocosmicus;s_Nitrosocosmicus sp. 013114705
	Mag_25c	87.79	0.65	2889762	64	70167	57.9	2927	GCA_040255825.1	d_Bacteria;p_Pseudomonadota;c_Alphaproteobacteria;o_Micropepsales; f_Micropepsaceae;g_Rhizomicrobium;s_
	Mag_31c	96.41	3.41	4200438	238	28578	66.4	3972	GCA_040255215.1	d_Bacteria;p_Pseudomonadota;c_Alphaproteobacteria; o_Reyranellales;f_;g_;s_
	Mag_32c	96.59	0	4105222	296	21243	67.9	3436	GCA_040255445.1	d_Bacteria;p_Planctomycetota;c_Phycisphaerae;o_Phycisphaerales; f_UBA1924;g_;s_
	Mag_34c	90.19	1.34	2022849	158	20347	64.3	2084	GCA_040255385.1	d_Bacteria;p_Pseudomonadota;c_Alphaproteobacteria;o_Sphingomonadales; f_Sphingomonadaceae;g_Sphingomicrobium;s_
	Mag_35c	97.15	1.28	3227804	246	22333	68.9	2954	GCA_040255345.1	d_Bacteria;p_Actinomycetota;c_Acidimicrobiia;o_Acidimicrobiales; f_JAAYBP01;g_JAFDWI01;s_

^*a*^ Completeness.

^*b*^ Contamination.

This study provides a valuable catalog of metagenomes and MAGs from enrichment microcosms, highlighting microbial communities with polyethylene biodegradation potential and laying a foundation for future research into microbial-driven environmental cleanup.

## Data Availability

Raw data files and MAGs are available under BioProject accession number PRJNA1081682. Metagenomic data are available under SRA accession numbers SRX23797258, SRX23797259, and SRX23797260. The NCBI Genome assembly numbers for the MAGs are provided in Table 1.
